# Is hyperdiploidy a favorable cytogenetics in adults with B‐lymphoblastic leukemia?

**DOI:** 10.1002/cam4.2255

**Published:** 2019-06-07

**Authors:** Zhining Chen, Yi Sun, Wei Xie, Sa A. Wang, Shimin Hu, Shaoying Li, Zhenya Tang, Gokce Toruner, L. Jeffrey Medeiros, Guilin Tang

**Affiliations:** ^1^ Department of Hematopathology The University of Texas MD Anderson Cancer Center Houston Texas; ^2^ Department of Pathology Affiliated Tumor Hospital, Guangxi Medical University Nanning China

**Keywords:** adult, B‐ALL, hyperdiploidy, pediatric, prognosis

## Abstract

Hyperdiploidy (chromosomal number 51‐65) is a common cytogenetic abnormality in pediatric patients with B‐lymphoblastic leukemia (B‐ALL) and belongs to the favorable cytogenetic subgroup. Hyperdiploidy in adult B‐ALL is much less common and its clinical significance has not been well studied. Among the 1205 patients with B‐ALL (1018 adults and 187 children) from our institution, 78 had a hyperdiploid karyotype, including 45 (4.4%) adults and 33 (17.6%) children (*P* < 0.0001). Among the patients with hyperdiploid B‐ALL, the adult group had a significantly inferior survival (similar to the patients with a normal karyotype) compared with the pediatric group (median survival: 42 months vs undefined, *P* = 0.0029). Hyperdiploidy in adults B‐ALL tended to more frequently harbor structural abnormalities (two or more) than children (53% vs 33%). Two or more structural abnormalities in a hyperdiploidy correlated with an adverse survival in adult patients (33 months vs undefined, *P* = 0.0008), similar to the survival of patients with a complex karyotype. We conclude that hyperdiploidy in adults with B‐ALL is less favorable and more commonly contains structural abnormalities comparing to pediatric patients. We suggest that hyperdiploidy with two or more structural abnormalities are best considered as a complex karyotype in adults with B‐ALL.

## 1 INTRODUCTION

Hyperdiploidy in B‐lymphoblastic leukemia (B‐ALL), defined by the presence of 51‐65 chromosomes, has been classified as a distinct subtype of B‐ALL in the World Health Organization classification of tumors of hematopoietic and lymphoid tissues.^1^ The most common numerical changes detected in hyperdiploid B‐ALL includes extra copies (often trisomies) of chromosomes 21, X, 14, 4, 10, and 17.^2^ Hyperdiploid B‐ALL comprises about 25%‐30% of pediatric B‐ALL cases^3^ and is often associated with a favorable prognosis in affected patients with a cure rate greater than 90%, especially when hyperdiploidy with simultaneous trisomies of chromosomes 4 and 10.^4^, ^5^, ^6^ Structural abnormalities are infrequent in hyperdiploid pediatric B‐ALL.^1^

In adults, in contrast, hyperdiploidy is much less common in B‐ALL, accounting for 7%‐8% of all adult B‐ALL cases.^1^, ^7^, ^8^ Due to the low frequency of hyperdiploidy in adults with B‐ALL, the prognostic significance and the specific chromosomal abnormalities (numerical and structural) of hyperdiploidy have not been well studied, although a few studies have suggested that hyperdiploidy in adults may not be as favorable as it in pediatric patients with B‐ALL.^7^

In this study, we reviewed 1205 B‐ALL patients from our institution (with majority being adult patients) and identified 78 patients with hyperdiploid B‐ALL. We compared the frequency, the cytogenetic features, and clinical outcomes of hyperdiploidy in B‐ALL in pediatric and adult patients.

## 2 MATERIALS AND METHODS

### 2.1 Case selection

We searched the database of the Department of Hematopathology at The University of Texas MD Anderson Cancer Center (MDACC) for all patients who were diagnosed with B‐ALL and managed at MDACC from year 2003 to 2018. We then narrowed down the list to cases of B‐ALL with 51‐65 chromosomes. Cases with monosomy(ies) and/or recurrent chromosomal translocations, such as t(9;22)(q34;q11.2)/*BCR‐ABL1*, t(v;11q23)/*KMT2A*, t(12;21)(p13.2;q22.1)/*ETV6‐RUNX1*, or t(1;19)(q23;p13.3)/*TCF3‐PBX1* were excluded. Clinical data, pathologic findings, and available mutation studies were reviewed. All samples were collected following institutional guidelines with informed consent in accord with the Declaration of Helsinki.

### 2.2 Cytogenetic analyses

Conventional chromosomal analyzes were performed on G‐banded metaphase cells prepared from unstimulated 24‐hour and 48‐hour bone marrow aspirate cultures using standard techniques. Twenty metaphases were analyzed in most of the cases and the results were reported using the International System for Human Cytogenetic Nomenclature (ISCN 2016). Based on the number of structural abnormalities in the hyperdiploid karyotype, cases were arbitrarily designated as hyper 0, hyper 1, hyper 2, and hyper 3, corresponding to 0, 1, 2, and 3 or more structural abnormalities, respectively.

### 2.3 Statistical analyses

The Chi‐square test or Fisher's exact test were applied for categorical variables. The Kaplan‐Meier method was used to estimate overall survival (OS) and disease‐free survival (DFS). OS is from the date of diagnosis until death from any cause, or censored at last follow‐up date for live patients, *P* < 0.05 was considered to be statistically significant.

## 3 RESULTS

### 3.1 Patients

A total of 1205 patients with B‐ALL were treated at our institution during the study period, including 187 children and 1018 adults. A total of 78 patients had a hyperdiploid karyotype, including 45 (4.4%) adults with a median age of 38 years (range, 18‐73 years) and 33 (17.6%) children with a median age of 6 years (range, 1‐16 years).

Among the patients with hyperdiploidy, adult patients showed significantly lower leukocytes comparing to children (median leukocytes: 2.2 × 10^9^/L vs 6.1 × 10^9^/L, *P* = 0.026). The children group showed more severe anemia comparing to the adult group (median hemoglobin: 10.1g/dL vs 9.1g/dL, *P* = 0.0069). No significant difference of platelet counts between adults and children (median: 62.5 × 10^9^/L vs 75 × 10^9^/L, *P* = 0.1437) (Table 2).

The treatments for pediatric patients with B‐ALL included different Children's Oncology Group protocols. The main treatments used for adults with B‐ALL was the hyper‐CVAD (hyperfractionated cyclophosphamide, vincristine, doxorubicin, dexamethasone alternating with high dose methotrexate and cytarabine) regimen, with or without rituximab. Of a note, among the 15 younger adults (age 18‐25 years), 11 received hyper‐CVAD, and four received augmented Berlin‐Frankfurt‐Munster (BFM) therapy. Besides, there were 18 patients (14 adults and four children) underwent allogeneic stem cell transplant (SCT), including eight adults underwent SCT after the first remission.

### 3.2 Cytogenetic features in hyperdiploidy

The most common numerical changes in adult patients included +21 (n = 30), an extra copy of chromosome X (n = 28), +14 (n = 26), +10 (n = 10), +6 (n = 22), and + 4 (n = 19). The least common numerical changes in adults were +16 (n = 2), +7 (n = 2), and + 3 (n = 4). The common structural changes in adult hyperdiploid B‐ALL cases were marker chromosome (n = 18) with unidentifiable genetic materials. The recurrent structural abnormalities included dup(1q) (n = 4), del(6q)(n = 3), add(14q) (n = 3), and del(9p) (n = 3).

**Figure 1:**
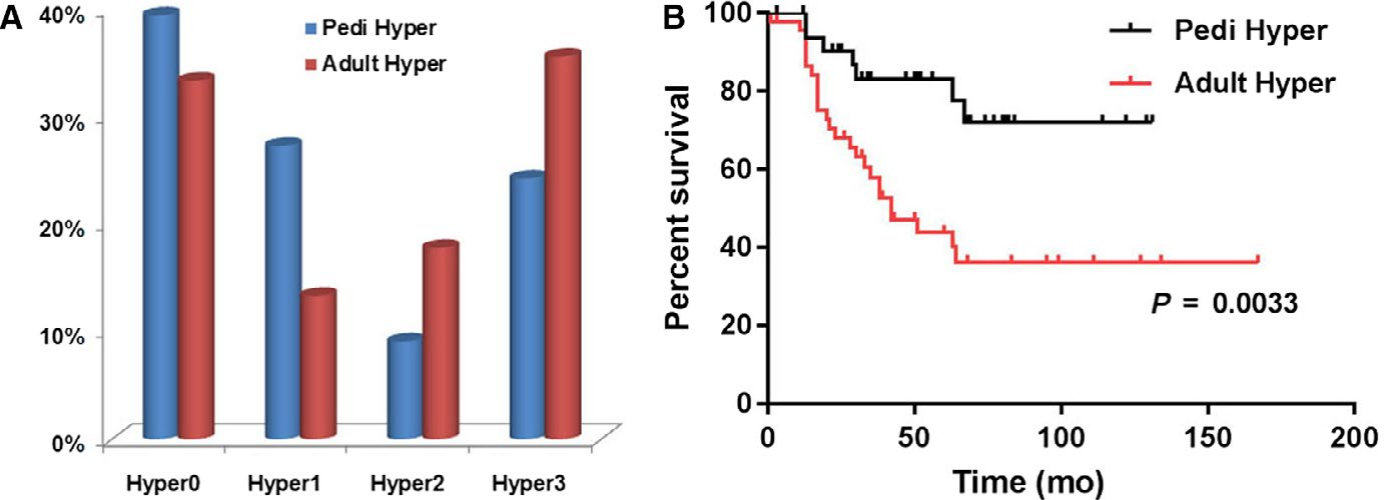
(A) The percentage of patients with different number of structural abnormalities (Hyper0‐3) in adult and pediatric B‐ALL; (B) comparison of overall survival of patients with hyperdiploidy in the adult and pediatric groups of B‐ALL

The number of patients with hyperdiploidy and no additional structural abnormalities (hyper0) was 15 in the adult group and 13 in the pediatric group. The number of patients with hyper1, hyper2, and hyper3 were 6, 8, and 16 in adults and 9, 3, and 8 in children, respectively (Figure 1A; Table 1).

**Table 1:** Features of hyperdiploidy in adult and pediatric patients

	Adult (n = 45)				Children (n = 33)			
Subgroups	Hyper0	Hyper1	Hyper2	Hyper3	Hyper0	Hyper1	Hyper2	Hyper3
Case No	15	6	8	16	13	9	3	8
Survival (median, mon)	Undefined	42	17	33	Undefined	Undefined	38.5	Undefined
*p* *		0.2362	0.0095	0.0025		0.3501	0.6437	0.2150
+4/+10	7	0	3	3	5	4	2	3

* Overall survival comparing to Hyper0.

### 3.3 Targeted next‐generation sequencing

Targeted next‐generation sequencing (NGS) studies using panels of genes commonly altered in hematopoietic neoplasms were performed in seven patients (two hyper0, one hyper1, one hyper2, and three hyper3) in adult group as a part of clinical workup, either with 28‐gene panel (n = 5) or 81‐gene panel (n = 2) as described previously.^9^ Two patients, one hyper0 and one hyper1, showed no mutation in any of the genes tested; one hyper0 showed *DNMT3A* and *PTPN11* mutation; one hyper2 showed *TP53* and *RAD21* mutations; three hyper3, one showed *CREBBP*, *TP53,* and *KRAS*, one *NRAS*, and one *TP53* mutations, respectively.

### 3.4 Cytogenetic subgroups and outcomes

The median follow‐up was 32 months (range, 0‐167 months). During the follow‐up, seven patients (six adults and one child) had persistent disease; 27 patients (18 adults and nine children) had relapse disease; and 44 patients (21 adults and 23 children) got into remission and had no relapse. Adults showed significantly higher frequency of refractory/relapsed disease comparing to children (53% vs 30%, *P* = 0.0427). By the end of the follow‐up, 25/45 adults died, including 19 died of disease (five with persistent disease and 14 with relapse disease) and six died of other causes; 7/33 children died of disease (one with persistent and six with relapsed disease).

Overall, adults with hyperdiploid B‐ALL had a significantly inferior overall survival (OS) than children (median survival: 42 months vs undefined, *P* = 0.0033, Figure 1B). In pediatric patients, the four subgroups (hyper0‐3), regardless of the number of structural abnormalities, showed comparable OS (Table 1). In contrast, adults patients with hyper1 had a comparable OS to patients with hyper0; however, patients with hyper2 and hyper3 showed a significantly inferior OS compared with patients with hyper0 (Table 1).

**Figure 2:**
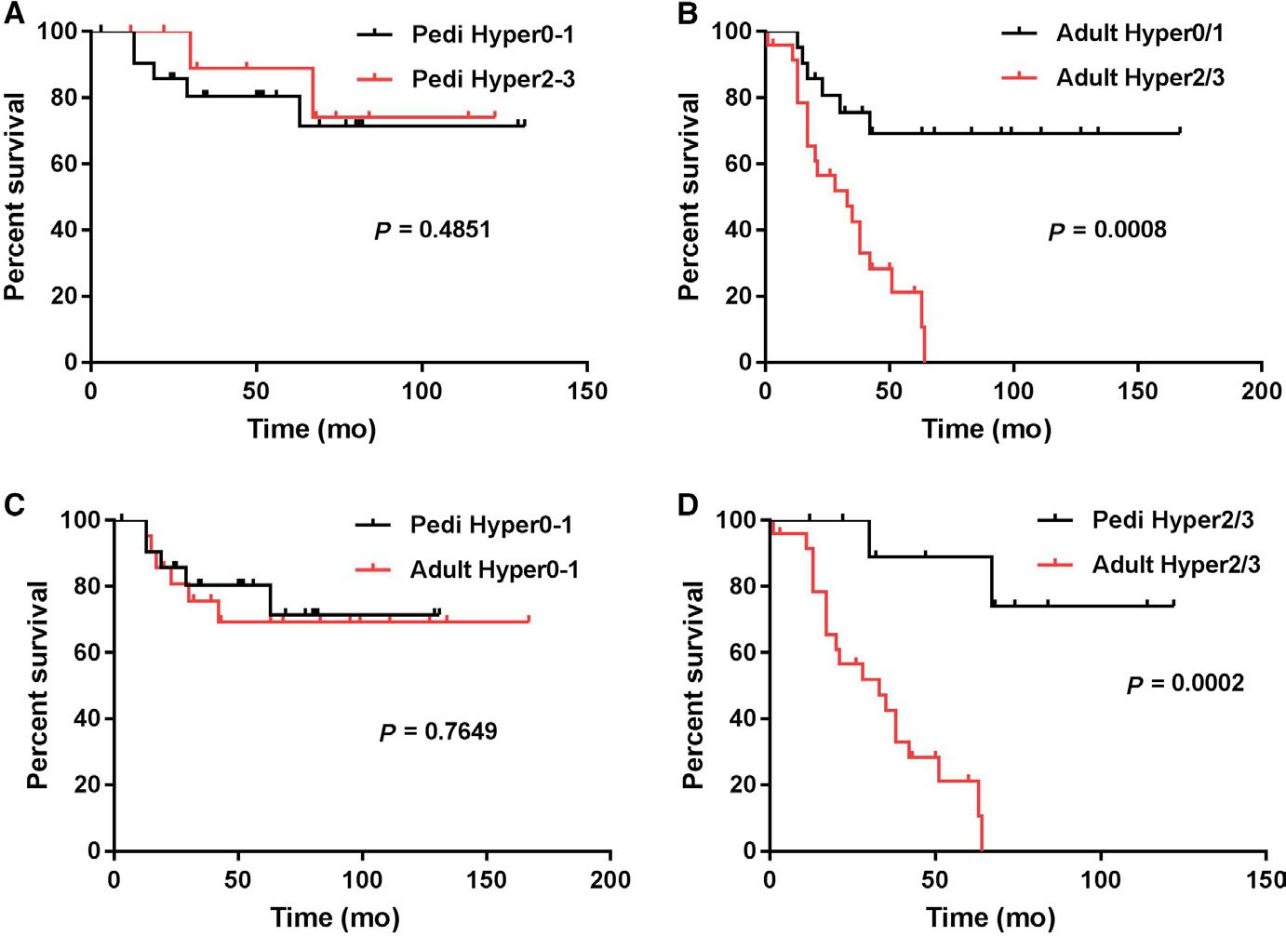
Overall survival comparison for patients with B‐ALL associated with hyperdiploidy. (A) Pediatric patients with hyper0/1 vs hyper2/3; (B) adults with hyper0/1 vs hyper2/3; (C) pediatric patients with hyper0/1 vs adults with hyper0/1; (D) pediatric patients with hyper2/3 vs adults with hyper2/3

Since hyper0 and hyper1 showed comparable OS in both the adult and pediatric groups, we combined patients with hyper0 and hyper1 as hyper0/1, and combined hyper2 and hyper3 as hyper2/3. In pediatric group, there were 22 patients with hyper0/1 and 11 with hyper2/3; in adult group, there were 21 patients with hyper0/1 and 24 with hyper2/3. The median age for patients with hyper0/1 and hyper2/3 was 4.5 and 8 years in pediatric group (*P* = 0.2377), 30 and 47 years in adult group (*P* = 0.1205, Table 2). In the pediatric group, patients with hyper2/3 showed a similar OS to patients with hyper0/1 (undefined vs undefined, *P* = 0.4815, Figure 2A). In contrast, in the adult group, patients with hyper2/3 showed an inferior OS compared with patients with hyper0/1 (undefined vs 33 months, *P* = 0.0008, Figure 2B). Interestingly, adult patients with hyper0/1 showed a similar OS as pediatric patients with hyper0/1 (undefined vs undefined, *P* = 0.7649, Figure 2C), but adults with hyper2/3 showed a poorer survival than children with hyper2/3 (33 months vs undefined, *P* = 0.0002, Figure 2D). Similarly, adults showed a significantly shorter DFS comparing to children (median DFS: 35 months vs undefined, *P* = 0.0134); adults with hyper2/3 showed a significantly shorter DFS comparing to adults with hyper0/1 (median DFS: 16 months vs undefined, *P* = 0.0022).

**Table 2:** Comparison the age and blood cell counts among patients with hyper0/1 and patients with hyper2/3

	Adult			Children			
	Total	Hyper0/1	Hyper2/3	Total	Hyper0/1		Hyper2/3
Case No	45	21	24	33	22		11
Age^a^ (years)	38 (18‐73)	30 (18‐69)	47 (19‐73)	6 (1‐16)	4.5 (1‐16)		8 (2‐15)
*p*		0.1205^+^			0.2377^+^		
WBC^a^ (x10^9^/L)	2.2 (0.5‐95)	2.1 (0.5‐50.9)	2.55 (0.6‐95)	6.1 (0.5‐24.3)	5.75 (0.5‐24.3)		6.1 (0.8‐18)
*p*		0.5335^+^		0.026*	0.4884^+^		
Hgb^a^ (g/dL)	10.1 (6.5‐15.5)	9.3 (8.1‐15.5)	10.2 (6.5‐15.4)	9.1 (4.5‐14)	9.6 (4.5‐14)	8.6 (5.4‐11.9)	
*p*		0.2276^+^		0.0069*	0.7659^+^		
Plt^a^ (x10^9^/L)	62.5 (12‐236)	77 (12‐236)	62 (14‐224)	75 (11‐344)	75 (11‐268)	100 (15‐344)	
*p*		0.6176^+^		0.1437*	0.8530^+^		

Abbreviations: WBC: white blood cells; Hgb: hemoglobin; Plt: platelets.

a Presented as median (range).

* *P* value of comparison between the adults with hyperdiploidy and the children with hyperdiploidy.

^+^ *P* value of comparison between the patients with hyper0/1 and patients with hyper2/3.

We compared the OS among the young adults (age 18‐25 years) who received augmented BFM (n = 4) and who received hyper‐CVAD (n = 11), there was no significant difference of OS among these two groups who treated differently (undefined vs 64 months, *P* = 0.4213).

**Figure 3:**
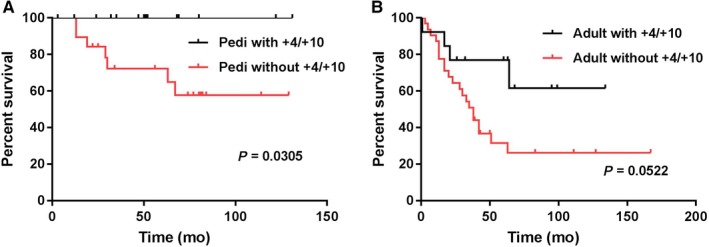
Comparision of overall survival among patients with B‐ALL associated with hyperdiploidy with or without simultaneous +4 and +10. (A) Pediatric patients; (B) adult patients

Simultaneous trisomy 4 and trisomy 10 (+4/+10) were detected in 14 of 33 (42%) pediatric patients and 13 of 45 (29%) adult patients (*P* = 0.2372). In the pediatric group, +4/+10 was distributed almost evenly in the four subgroups. However, in the adult group, +4/+10 was detected more frequently in the hyper0 subgroup than in the other three subgroups (Table 1). The presence of +4/+10 was associated with a superior OS in pediatric (undefined vs undefined, *P* = 0.0305, Figure 3A) as well as adult patients (undefined vs 38 months, *P* = 0.0522, Figure 3B).

**Figure 4:**
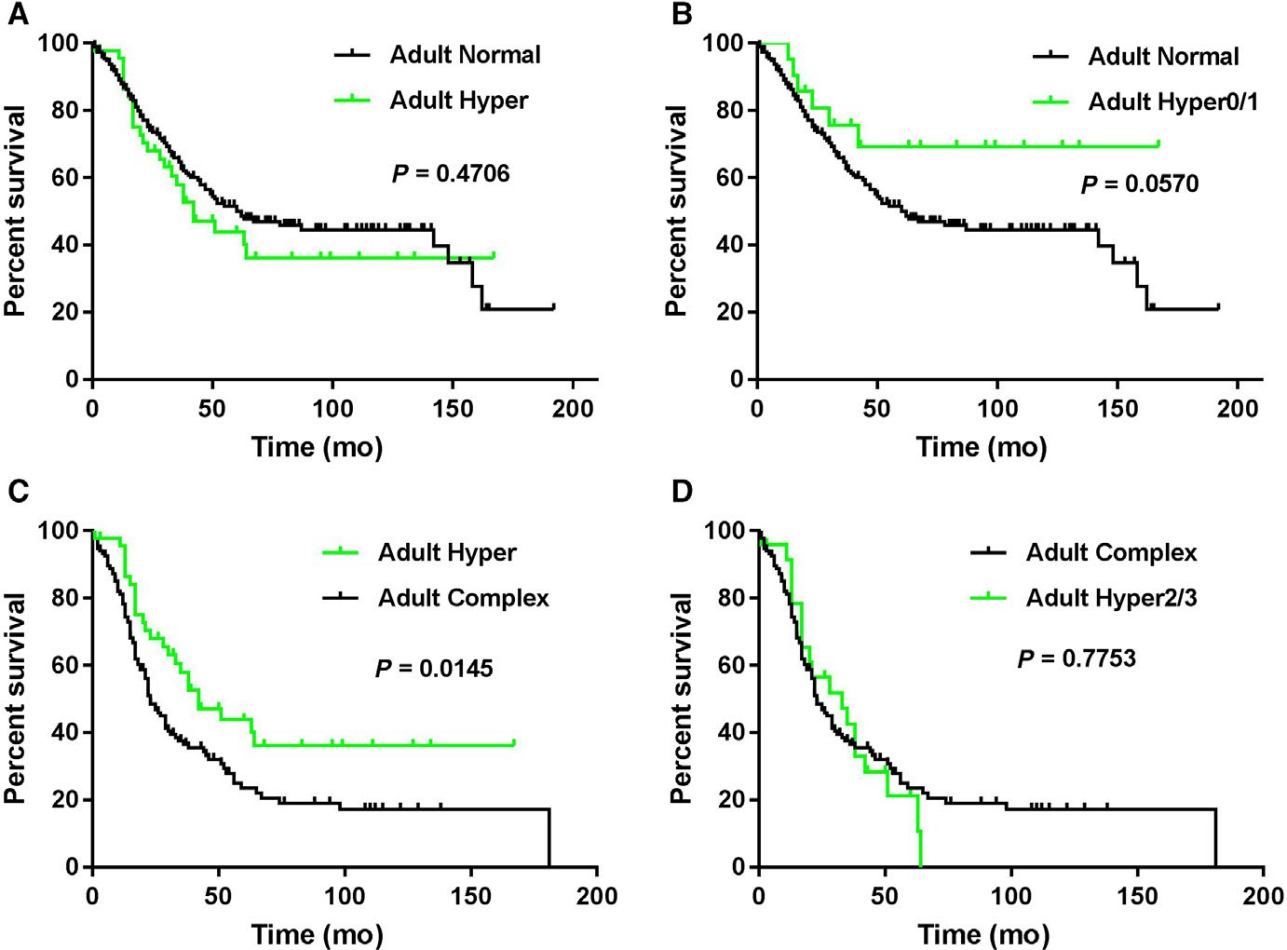
Comparison of overall survival in adults with B‐ALL and hyperdiploidy, normal and complex karyotype. (A) Hyperdiploidy vs normal karyotype (n = 199); (B) hyper0/1 vs normal karyotype; (C) hyperdiploidy vs complex karyotype (n = 135); (D) hyper2/3 vs complex karyotype

In children with B‐ALL, the presence of hyperdiploidy appeared to be associated with a better OS compared to children with a normal karyotype (undefined vs 130 months, *P* = 0.0682). In adults with B‐ALL, the presence of hyperdiploidy was associated with an OS similar to those with a normal karyotype (median survival: 42 vs 62 months, *P* = 0.4706, Figure 4A). However, patients with hyper0/1 showed better OS than those with a normal karyotype (undefined vs 62 months, *P* = 0.0570, Figure 4B).

In adult B‐ALL patients, the presence of hyperdiploidy predicted a superior OS compared to patients with a complex karyotype (median survival: 42 vs 23 months, *P* = 0.0145, Figure 4C). However, patients with hyper2/3 had an OS similar to patients with a complex karyotype (33 vs 23 months, *P* = 0.7753, Figure 4D).

## 4 DISCUSSION

B‐ALL has a very good prognosis in children, but is much less favorable in infants or older adults. One important factor that contributes to prognosis is the differences in cytogenetics findings associated with different age groups: for example, a high frequency of favorable cytogenetics groups such as hyperdiploidy and t(12;21)(p13;q22.1)/*ETV6‐RUNX1* in children versus a high frequency of unfavorable cytogenetics such as t(11q23;v)/*KMT2A* in infants and t(9;22)(q34;q11.2)/*BCR‐ABL1*, hypodiploidy and/or a complex karyotype in adults.^1^ Studies have shown that age itself might not be a significant prognostic factor in adult B‐ALL, but the presence of unfavorable cytogenetics.^10^

Hyperdiploidy is a common cytogenetic finding in children with B‐ALL and is associated with a very favorable prognosis.^1^, ^11^ In this study, we retrospectively reviewed 1,018 adult patients with B‐ALL and identified 45 (4.4%) with hyperdiploidy. At the same time, we also identified 33 (17.6%) of 187 pediatric patients with hyperdiploid B‐ALL. The frequency of hyperdiploidy is slightly lower in both pediatric and adult study group in our cohort compared with the published data, about 25% for children and 7%‐8% for adults.^1^, ^2^, ^7^, ^8^ These differences may be attributable to the more strict criteria for hyperdiploidy applied in this study as we excluded all patients with recurrent translocations and/or monosomies.^8^ There also may be referral bias as well, as patients with high risk B‐ALL are more likely to seek secondary treatment options at our institution.

Numerical chromosomal gains are nonrandom in hyperdiploidy, with extra copies (usually trisomies) of chromosomes 21, X, 14, and 4 being detected most often in pediatric patients.^12^ In this study we showed a similar pattern in adult patients, with extra copies of chromosomes 21, X, 14, 10, and 4 being the most common numerical abnormalities. One difference was trisomy 6, which was very common in adults, but not common in pediatric B‐ALL patients. As has been observed in children,^13^, ^14^, ^15^ simultaneously presence of +4/+10 was also associated with a superior outcome in adults in this study. Extra copies of chromosomes 1, 2, and 3 have been reported to be as the least common in children hyperdiploid B‐ALL,^12^ while extra copies of chromosomes 3, 7, and 16 are the least common in adults.

Structural chromosomal abnormalities are commonly detected in hyperdiploidy in both adult and pediatric B‐ALL patients, 66% and 61%, respectively in this study, in line with what reported in previous studies.^11^, ^13^, ^16^, ^17^ However, two or more structural abnormalities were more commonly detected in adults than in children (53% vs 33%). Unlike numerical changes, structural abnormalities tend to be random. dup(1q)/gain of *CKS1B*, del(6q)/deletion of *MYB*, and del(9p)/deletion of *CDKN2A*, which are common in lymphoid neoplasms, were detected at a low frequency in adult B‐ALL patients with hyperdiploidy.

In the adult B‐ALL group in this study, patients with hyperdiploidy showed a similar overall survival to those with a normal diploid karyotype, similar to what reported previously.^8^ Adults with B‐ALL with hyperdiploidy also had a worse outcome comparing to children with hyperdiploid B‐ALL. In addition, structural abnormalities appear to impact survival differently in adults vs children. We found that structural abnormalities (regardless of the number) in a hyperdiploidy did not impact the favorable outcome in children, consistent with previous findings.^11^ However, this was not true in adult patients. Adult group with 0‐1 structural abnormalities had a favorable survival similar to pediatric group, whereas two or more structural abnormalities in adults adversely affected outcomes, with a survival similar to a complex karyotype. Thus we propose that the number of structural abnormalities in hyperdiploidy B‐ALL is important in adult patients: hyperdiploidy with no or one structural abnormality could be considered as a “classical” hyperdiploid karyotype which is often associated with a superior outcome, but we suggest that hyperdiploidy with two or more structural abnormalities is better classified as a complex karyotype. As was discussed above, the presence of ≥2 structural abnormalities was more common in adults than children. These findings, in conjunction with other studies, suggest that the frequency of structural abnormalities/complex karyotype in a hyperdiploid karyotype increases with age in ALL patients and has an adverse impact on prognosis.^18^

Due to many cases were from earlier years when NGS was not available clinically, only a small subset of cases had NGS mutation data available. However, the data indicated that *TP53* mutation was commonly detected in patients with hyper2/3 (in three of four patients), but not in patients with hyper0/1 (in zero of three patients). High frequency of *TP53* mutation also supports that hyper2/3 is a high‐risk cytogenetic group in adult B‐ALL patients. Unfortunately, we do not have the NGS data from pediatric group.

In summary, hyperdiploidy occurs infrequently in adult B‐ALL and appears to have different prognostic implications as compared with childhood B‐ALL. In general, adults with hyperdiploidy more often have two or more structural abnormalities. In addition, hyperdiploidy with one or less structural abnormality in adults is often associated with a superior outcome and should be considered as “classic” hyperdiploidy; however, hyperdiploidy with two or more structural abnormalities is often associated with a worse prognosis in adults and should be classified as a complex karyotype.
